# Myelodysplasia Syndrome, Clonal Hematopoiesis and Cardiovascular Disease

**DOI:** 10.3390/cancers13081968

**Published:** 2021-04-19

**Authors:** Camilla Bertuzzo Veiga, Erin M. Lawrence, Andrew J. Murphy, Marco J. Herold, Dragana Dragoljevic

**Affiliations:** 1 Division of Immunometabolism, Baker Heart and Diabetes Institute, Melbourne, VIC 3004, Australia; camillabertuzzo.veiga@baker.edu.au (C.B.V.); andrew.murphy@baker.edu.au (A.J.M.); 2 Department of Anatomy and Physiology, University of Melbourne, Parkville, Melbourne, VIC 3010, Australia; 3 Walter and Eliza Hall Institute of Medical Research, 1 G Royal Parade, Parkville, Melbourne, VIC 3052, Australia; lawrence.e@wehi.edu.au (E.M.L.); herold@wehi.edu.au (M.J.H.); 4 Department of Medical Biology, University of Melbourne, Parkville, Melbourne, VIC 3052, Australia; 5 Department of Diabetes, Department of Immunology, Monash University, Clayton, VIC 3004, Australia; 6 Baker Department of Cardiometabolic Health, University of Melbourne, Melbourne, VIC 3052, Australia

**Keywords:** myelodysplasia syndrome, clonal hematopoiesis and indeterminate potential (CHIP), DNMT3A, TET2, P53, ASXL1, JAK2, hematopoietic stem cell (HSC), cardiovascular disease

## Abstract

**Simple Summary:**

The development of blood cancers is a complex process that involves the acquisition of specific blood disorders that precede cancer. These blood disorders are often driven by the accumulation of genetic abnormalities, which are discussed in this review. Likewise, predicting the rate of progression of these diseases is difficult, but it appears to be linked to which specific gene mutations are present in blood cells. In this review, we discuss a variety of genetic abnormalities that drive blood cancer, conditions that precede clinical symptoms of blood cancer, and how alterations in these genes change blood cell function. Additionally, we discuss the novel links between blood cancer development and heart disease.

**Abstract:**

The development of myelodysplasia syndromes (MDS) is multiphasic and can be driven by a plethora of genetic mutations and/or abnormalities. MDS is characterized by a hematopoietic differentiation block, evidenced by increased immature hematopoietic cells, termed blast cells and decreased mature circulating leukocytes in at least one lineage (i.e., cytopenia). Clonal hematopoiesis of indeterminate potential (CHIP) is a recently described phenomenon preceding MDS development that is driven by somatic mutations in hemopoietic stem cells (HSCs). These mutant HSCs have a competitive advantage over healthy cells, resulting in an expansion of these clonal mutated leukocytes. In this review, we discuss the multiphasic development of MDS, the common mutations found in both MDS and CHIP, how a loss-of-function in these CHIP-related genes can alter HSC function and leukocyte development and the potential disease outcomes that can occur with dysfunctional HSCs. In particular, we discuss the novel connections between MDS development and cardiovascular disease.

## 1. Introduction

Myelodysplasia syndrome (MDS) is primarily a disease of the elderly, with an average age of diagnosis at 70 years of age. MDS is a myeloid neoplasm that originates in hematopoietic stem cells (HSCs), resulting in dysregulation of hematopoiesis, bone marrow (BM) dysplasia, peripheral blood cytopenia (reduced blood cells) and predisposition to the development of acute myeloid leukemia (AML) [1,2]. Furthermore, patients with MDS generally die of conditions related to MDS, such as increased bleeding or susceptibility to infections due to the cytopenia’s, or the progression to AML. However, patients can also have co-morbidities related to ageing, such as cardiovascular disease (CVD), which may have a compounding effect on MDS related complications [3,4].

In this review, we discuss the disorders that precede MDS development, the vast number of genetic abnormalities that can cause MDS and how the dysfunction of MDS-related genes alters HSC activity and the hematological consequences. Additionally, we discuss the novel association of increased CVD in MDS patients.

## 2. MDS Pathophysiology

**Figure 1:**
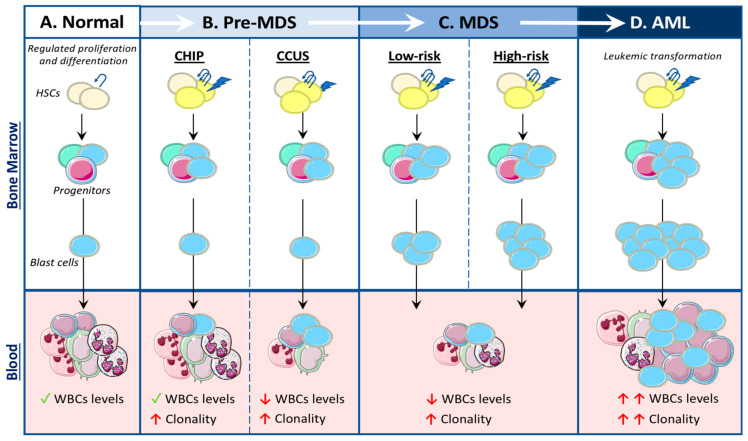
Transformations from normal hematopoiesis to acute myeloid leukemia (AML) development. (**A**) Normal: Normal hematopoiesis is highly regulated, whereby hematopoietic stem cells (HSCs) can self-renew and differentiate into progenitor cells. Progenitor cells differentiate into immature blood cells (blast cells) and then into mature white blood cells (WBCs). (**B**) Pre-MDS (myelodysplasia syndrome): Clonal hematopoiesis of indeterminate potential (CHIP) occurs when a HSC acquires a somatic mutation resulting in a proliferative advantage; giving rise to a mutated clone. While the levels of WBCs do not change, there are mutated clonal WBCs detected in the blood. Clonal cytopenia of undetermined significance (CCUS) presents with CHIP, as well as with cytopenia. (**C**) MDS: The development of significant bone marrow (BM) blast cells, BM dysplasia and cytopenia is usually associated with at least 1 genetic abnormality (either a gene mutation or chromosomal defect). MDS is considered low risk (transformation potential) when blast cell numbers are relatively low, and high risk when BM dysplasia is advanced. (**D**) AML: A leukemic transformation occurs that results in excessive BM expansion, BM dysplasia and pathological WBC production.

MDS results in disordered hematopoiesis as a consequence of genetic abnormalities in HSCs. Hematopoiesis is a tightly regulated and hierarchically structured process of blood production [5] (Figure 1A). HSCs give rise to all blood cells, and hence are classified as having multilineage potential. Generally speaking, HSCs can differentiate into either common myeloid progenitors (CMPs), which give rise to myeloid cells such as monocytes, neutrophils and platelets, or can differentiate into common lymphoid progenitors (CLPs) which generate B and T-cells.

The development of MDS is a multiphasic process. Firstly, an “initiating mutation”, or other genetic abnormality, is acquired in an HSC, which primes the cell but is not sufficient to induce clinical hematological disease. Subsequently, an additional mutation or multiple mutations are acquired in either the HSC or downstream in the myeloid progenitors, resulting in a proliferative advantage and an impaired differentiation capacity. These mutated cells cause an increase in immature blood cells (i.e., blast cells) and loss of mature circulating leukocytes from one or more lineages (i.e., Cytopenia) due to this differentiation block (Table 1 and Figure 1) [6]. Lastly, MDS can convert to AML when there is a leukemic transformation, drastically increasing HSCs, progenitors, and blast cells; circulating leukocytes; and reducing blood cell differentiation [7]. The factors that drive the acquisition of MDS-related genetic mutations are incompletely understood [8]. Conventional chemoradiotherapy has been shown to cause a therapy- or treatment-related MDS (t-MDS/t-AML) in patients receiving treatment for other malignancies [9,10,11]. However, for the vast majority of patients, the cause of genetic abnormalities driving MDS is less understood [8,12].

### 2.1. MDS Precursor Conditions

#### 2.1.1. CHIP

Clonal hematopoiesis of indeterminate potential (CHIP) is characterized by the acquisition of somatic mutations in HSCs which provide a competitive advantage over healthy HSCs, leading to an increase in the number of mutated HSCs, progenitors and their mature progeny (Figure 1B) [13,14,15,16,17,18]. While there is no change in total circulating white blood cells in CHIP, it results in a progressive clonal-specific expansion of WBCs, and ultimately an increased risk of hematological cancers. Additionally, there is significant overlap between mutations identified in CHIP and MDS. The most common genetic mutations affect DNA methyltransferase 3A (DNMT3A), Tet methylcytosine dioxygenase 2 (TET2) and additional sex combs like 1 (ASXL1). Due to the high mutational overlap between CHIP and MDS, CHIP is considered to be a precursor to MDS.

Age is the most clearly defined risk factor for the development of CHIP, with less than 5% of people under the age of 60 carrying a CHIP mutation, and up to 18% of people over the age of 90 testing positive for a CHIP mutation [17,19]. Interestingly, people with CHIP can remain stable with a low variant allele frequency (VAF) and with no clinical symptoms. People with CHIP may go on to develop a myeloid neoplasm such as MDS or AML, or may not develop any malignant disease. However, what drives the transformation from CHIP to MDS/AML is not well-known, but certain mutations have been linked with stronger chance of malignant transformation than others. These are mentioned in this review. Interestingly, whilst mutations in CHIP are strongly linked to hematological cancers such as MDS/AML, it was recently described that people with CHIP were also at increased risk of death due to atherosclerotic CVD [14,17,19,20].

#### 2.1.2. CCUS and ICUS

When a CHIP mutation is associated with blood cytopenia without, or with very little, change in BM blast numbers or BM dysplasia, it is termed clonal cytopenia of undetermined significance (CCUS; Figure 1B and Table 1) [1]. VAF in CCUS tends to be extremely high, usually greater than 30%, and has also been linked to a higher risk of MDS and AML progression [21,22]. One study calculated a 70% chance of malignant progression in patients with CCUS within 4 years of diagnosis [22]. Furthermore, idiopathic cytopenia of undetermined significance (ICUS) refers to situations when cytopenia is the only symptom identified without evidence of clonal cells or MDS morphology. This is also considered a pre-MDS condition but has a relatively low risk of MDS progression compared to CHIP and CCUS [22]. Therefore, diagnosing these conditions requires multiple parameters, and just the presence of a mutation in the peripheral blood alone cannot determine whether it is CHIP/CCUS (pre-MDS) or MDS without also assessing blood counts and BM characteristics (Table 1).

## 3. Genetic Abnormalities in MDS Development

MDS is defined as hematological abnormalities that result in increased immature cells (blast cells) and cytopenia in one or more lineages (Figure 1C and Table 1). Multiple genetic mutations and chromosomal abnormalities have been identified to cause MDS, and this diversity in driver mutations is responsible for the phenotypic heterogeneity observed in MDS (Table 2). The genes that are most commonly mutated in MDS are involved in a variety of cellular regulatory functions, including DNA methylation, RNA-Splicing, DNA transcription, histone modification, signal transduction and the cohesion complex subunits [7,12,23,24]. Generally, mutations in epigenetic and RNA-splicing processes are termed “driver mutations”, as it is these mutations that tend to dictate what type of clone will outgrow and outcompete (also termed “clonal dominance”) [25,26,27]. This is combined by other mutations that contribute to clonal proliferation and progression, which eventually lead to MDS and/or AML.

### 3.1. Somatic Mutations

At the onset of the clinical disease, MDS patients will exhibit an average of two or three mutations. As many as 10 additional genetic mutations can be acquired as the disease progresses [28]. While MDS can occur from alterations in over a hundred genes, mutations in only six genes account for the majority of MDS cases: TET2, ASXL1, DNMT3A, the splicing factor 3b, subunit 1 (*SF3B1*), the serine and arginine-rich splicing factor 2 (*SRSF2*) and the RUNX family transcription factor 1 (*RUNX1*) [6,26,29]. Interestingly, somatic mutations in differing genes are associated with diverse MDS stages, disease progression and clinical outcome [30,31]. For example, mutations in epigenetic regulators, e.g., *TET2* or *DNMT3A*, can remain in the CHIP phase for many years, while modifications in spliceosome genes (e.g., *SF3B1* and *SRSF2*) have a more rapid transformation to MDS [22].

#### 3.1.1. Epigenetic Regulators

*DNMT3A*, *TET2*, *ASXL1* and the enhancer of zeste 2 polycomb repressive complex 2 subunit (EZH2) are the most frequently mutated epigenetic regulators in MDS [24,32,33,34]. *DNMT3A* and *TET2* regulate DNA methylation/demethylation respectively, which is particularly important in influencing stem-cell renewal, function and differentiation [20,35,36]. *DNMT3A* methylates DNA by transferring methyl groups to specific CpG regions in DNA. Mutations in *DNMT3A* are found in up to 13% of all MDS subtypes, and while mutations occur across the gene, there is strong enrichment for the R882 mutation which affects the catalytic domain of the protein [24,32]. *DNMT3A* *R882* mutations tend to occur early in disease progression (i.e., in CHIP) and are associated with a rapid progression to AML and decreased survival [24,32]. On the other hand, mutations in *TET2*, which demethylates DNA by converting 5-methylcytosine (5 mc) to 5-hydroxy-methylcytosine (5 hmc), are associated with a mild clinical prognosis [31,33,34]. Interestingly, if a TET2 mutation is acquired as the second or later mutation in the development of MDS, it does not appear to stimulate MDS progression further, but does promote monocytic differentiation [33]. TET2 mutations can occur throughout the entire gene and almost always result in a loss-of-function [35]. It is found in up to 25% of all MDS cases and is also the most commonly mutated gene in low-risk MDS [31]. However, mutations in ASXL1, which modulates post-translational histone modifications, are strongly associated with disease progression, poor prognosis and lower survival rates [31,37,38]. Similarly, mutations in EZH2, a histone methyltransferase, are also linked with poor clinical outcome [31,38,39].

#### 3.1.2. RNA Spliceosome

The most common RNA-splicing genes mutated in MDS are *SF3B1*, *SRSF2* and U2-complex auxiliary factor 1 (*U2AF1*); and zinc finger CCCH-type RNA binding motif and serine/arginine-rich 2 (*ZRSR2*) [12,23,40,41]. The most common *SF3B1* mutation observed is a lysine to glutamic acid substitution at codon 700 (K700E); however, mutations in the conserved amino acids 622, 625, 662 and 666 are also documented to a lesser degree [23,42,43]. Interestingly, *SF3B1* mutations are more commonly associated with a low-risk type of MDS called refractory anemia with ring sideroblasts (RARS) [23,42]. In contrast, somatic mutations in *U2AF1*, most frequently mutated in sites S34 and Q157, are associated with MDS to AML progression and worse clinical outcome [23,31]. Additionally, *SRSF2* mutations, which nearly exclusively occur at proline 95 (P95H), are also linked with poor survival outcome [6,23,44]. Indeed, *SRSF2* mutations are found in 14% of MDS patients and up to 47% of patients with another form of leukemia, chronic myelomonocytic leukemia (CMML) [6,44]. Additionally, *U2AF1* and *SRSF2* mutations also lead to a downregulation of *EZH2* which further drives MDS and AML disease progression [39]. Interestingly, while mutations in *U2AF1* and *SRSF2* are both common in MDS, they are mutually exclusively suggestive of a lethal interaction [41].

#### 3.1.3. DNA Transcription

Mutations in *RUNX1* and the tumor protein P53 (*TP53*) genes, which are involved in DNA transcription, are well-known to be associated with poor prognosis of disease outcome in MDS/AML [31,38,45,46]. RUNX1, also known as acute myeloid leukemia 1 (AML1) protein, is well-known to influence HSC differentiation and hematopoiesis, and therefore, a loss-of-function mutation in RUNX1 drastically increases disease progression and leukemia transformation [31,38,45]. The most common mutation, *D171N*, results in increased HSC self-renewal, impeded differentiation and drastic dysplasia [45]. Similarly, P53 is involved in cell-cycle arrest, proliferation, cell senescence, apoptosis and differentiation [47]. Loss-of-function P53 mutations are commonly associated with the high-risk MDS, and these mutations carry one of the worst disease prognoses in MDS/AML [31,38,46].

#### 3.1.4. Other Genes

MDS patients can also carry mutations in the Kirsten rat sarcoma viral oncogene homolog (KRAS) and the neuroblastoma RAS viral oncogene homolog (NRAS), which are involved in MAPK signal transduction pathway. While they occur in lower frequencies than the previous genes mentioned in this review (~5% of MDS), KRAS/NRAS mutations often occur during the transition of MDS to AML and are associated with reduced survival rates [31,38]. Mutations in Janus Kinase 2 (JAK2) play a crucial role in signal transduction; moreover, protein expression is also found in MDS but at lower frequencies (~5% of MDS). Furthermore, the subunits which comprise the cohesion complex involved in transcriptional co-activation during cell division can also be mutated and are generally also associated with high-risk MDS and AML progression [48]. These include the *SMC3*, *SMC1A*, *RAD21* and *STAG2* genes [48].

### 3.2. Germline Mutations

While MDS generally develops in the elderly, there is a growing body of evidence showing that a portion of the population appears to have a genetic predisposition to MDS [1,49,50]. In these cases, the germline mutation is the primary mutation and the disease progresses with the accumulation of further somatic mutations. A total of 15% of all MDS cases are thought to arise from an initial germline mutation [49]. While alterations in genes such as *CEBPA*, *DDX41*, *ETV6*, *GATA2* and *RUNX1* are rare, they are also associated with MDS and/or AML development [49]. Additionally, several inherited disorders increase the risk of MDS development [49]. These include Fanconi anemia (*FANC* genes), Shwachman–Diamond syndrome (*SBDS* gene), Li-Fraumeni Syndrome (*TP53*), Diamond–Blackfan anemia (*GATA1/RPS19*), Dyskeratosis congenita and other telomerase complex disorders to name a few [7,12,49]. Patients with Tatton–Brown–Rahmann Syndrome caused by germline mutations in DNMT3a may also be at an increased risk of developing MDS/AML; however, this syndrome was only recently described, and there is limited longitudinal data, as all of the patients are still very young [51,52].

### 3.3. Chromosomal Abnormalities

Chromosomal abnormalities account for about half of all MDS cases and are even more common in t-MDS. These abnormalities include chromosomal loss, amplification, gain and balanced translocations. Additionally, genetic mutations involved in DNA repair or DNA methylation can cause chromosomal instability and consequently also confer a chromosomal defect. Indeed, unbalanced DNA methylation changes can induce chromosomal rearrangements and are frequently observed in a plethora of oncogenic diseases [12,53].

The most common chromosomal abnormality in MDS is the deletion of the long arm of chromosome 5 (5q), del (5q), and can be found in up to 15% of all MDS cases [54,55]. Patients with this sole chromosomal abnormality tend to have a relatively mild case of MDS that does not stimulate malignant transformation to AML. However, if this deletion occurs with either complex karyotypes (that is, at least three chromosomal defects) or with a TP53 gene mutation, an increased risk of transformation to AML is observed, as well as a poor prognosis, resulting in worse clinical outcomes [54,55,56].

Other more common chromosomal deletions that can lead to MDS include partial or total loss of chromosome 7; del (7q) and chromosome 20q deletion; del (20q) and chromosome 17 deletion; or del (17q). Del (7q) is more common in t-MDS (~50% of patients), and less so in de novo MDS (~10%), but regardless of MDS type, patients with this chromosomal abnormality have a poor prognosis [12,57]. Similar to del (5q), del (20q) is generally observed in low-risk MDS patients, unless it is coupled with somatic mutations such as ASXL1 and U2AF1 [58]. Comparably, del (17q) is frequently associated with TP53 mutations and is consequently considered a very high-risk type of MDS, but it is also very rare occurring in about ~1 of all MDS cases [55].

In addition to chromosomal deletions, MDS can also be driven by Trisomy 8, complex karyotypes and a list of other rare defects, including chromosomal translocations [54]. Trisomy 8 tends to appear late and is detected commonly in the setting of AML. It is associated with many mutations, including RUNX1, ASXL1 and transcription factor genes. Furthermore, MDS can occur as a consequence of complex karyotypes [54]. Complex karyotypes occur when there are at least three cytogenetic abnormalities and is often associated with gene mutations such as TP53 [54]. Essentially, MDS is an extremely cytogenetically unstable disease that is associated with many chromosomal abnormalities that can be detected across the entire karyotype.

### 3.4. MDS Progression to AML

AML occurs when there is a leukemic transformation from MDS that results in excessive immature WBC production (Figure 1D). The progression from MDS to AML can either be linear, with the expansion of one mutant clone, or by branching of multiple different mutant clones [28]. Linear expansion can also occur when subclones evolve from the first clone but take over the original clone. Contrarily, one or more clones can outcompete the existing one or more clones to generate an expansion of multiple clones [28]. Clinicians utilize the International Prognostic Scoring System (IPSS), the Revised International Prognostic Scoring System (IPSS-R) and the Low-Risk Prognostic Scoring System (LR-PSS) to stratify the risk of MDS to AML progression [59]. These tools classify MDS as either low risk or high risk, thus allowing clinicians to tailor their patients’ therapeutic goals relative to the risk of AML transformation. The risk of transformation to AML is determined by factoring in percentage of BM blasts, any karyotype abnormalities and the number of blood cytopenia’s. While these formulas are the best scoring systems at present, they still do not predict AML progression with certainty. Furthermore, evidence suggests that identifying the precise mutations to refine these scoring systems would enhance prognostic accuracy [59]. Interestingly, different mutations in the same gene can also predict differential impact AML on progression and survival. For example, DNMT3A mutations at R882 have been shown to have the most severe AML transformation potential, whereas other DNMT3A mutations are far less likely to progress [60].

## 4. The Dysfunction of CHIP-Related Genes Alters HSC Function

Somatic mutations in CHIP-related genes predispose individuals to the development of MDS and AML, and, as such, CHIP precedes hematologic neoplasms such as MDS. While patients with CHIP do not display obvious hematological changes, such as changes in absolute WBC levels, these mutated clones have a divergent epigenetic landscape and an altered methylome which can alter HSC lineage priming (i.e., unbalanced expression of myeloid, lymphoid and/or erythroid genes), proliferation frequency, cellular renewal and/or differentiation capacity. Undeniably, changes in DNA methylation have been significantly implicated in all three disease states, namely CHIP, MDS and AML, highlighting the importance of epigenetic regulation in normal hematopoiesis [14,17,19,24]. Indeed, mutations in the epigenetic modifiers *DNMT3a* and *TET2* are the most potent drivers of CHIP present in around 95% of cases [61,62]. Here we discuss the cellular and hematopoietic consequences of a loss of function of the top five mutated genes in CHIP.

### 4.1. DNMTs

DNA methylation is catalyzed by a family of DNMT enzymes consisting of *DNMT1*, *DNMT3A* and *DNMT3B*, whereby *DNMT3A* and *DNMT3B* perform de novo methylation in unmethylated DNA [63,64]. While mutations in DNMT1 are rarely observed in MDS or CHIP, mutations in *DNMT3A* are frequently detected in CHIP.

Mutant *DNMT3a* is the most commonly identified driver of CHIP, irrespective of age, across multiple studies [61,62,65,66]. Intriguingly, while mutant *DNMT3a* is the most frequently identified gene in patients with CHIP, it does not strongly alter blood-cell composition in people [62]. Mutant *DNMT3a* is pervasive in the blood of middle-aged people who are otherwise completely healthy, making it difficult to determine that pathogenicity of mutant *DNMT3a* driven CHIP [61]. However, not all mutations in *DNMT3a* are made equal, and a recent study has revealed that people with *DNMT3A*-R882-driven CHIP have significantly higher VAF frequencies than people with non-R882 mutations, suggesting that different mutations confer different outcomes [65]. *DNMT3A* has been shown to play a role in HSC differentiation, and this could explain why mutant *DNMT3a* is so abundant in CHIP.

**Figure 2:**
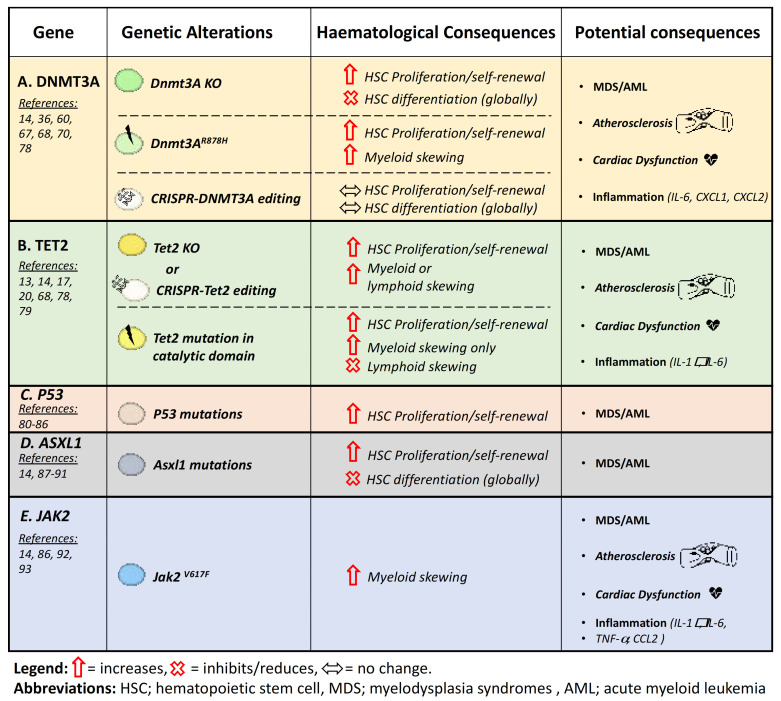
Hematopoietic and disease consequences of the top 5 genes mutated in CHIP. (**A**) DNMT3A: KO, single spot loss-of-function mutation and CRISPR editing studies [14,36,60,67,68,70,78]. (**B**) TET2: KO, CRISPR editing or mutation in the catalytic domain only studies [13,14,17,20,68,78,79]. (**C**) P53: studies utilizing P53 loss-of-function mutations [80,81,82,83,84,85,86]. (**D**) ASXL1: studies utilizing loss-of-function mutations [14,87,88,89,90,91]. (**E**) JAK2: studies reporting the V617F mutation [14,86,92,93]. KO, Knock Out; HSC, hematopoietic stem cell.

HSCs carrying a *DNMT3A* mutation generally have increased cellular renewal capacity and reduced differentiation (Figure 2A) [36,67,68]. This is evidenced by both in vitro and in vivo observations—a significant decline in differentiation capacity with HSC serial BM transplantations presenting with undifferentiated HSC accumulation in the BM [36], as well as reduced colonies formed with colony forming unit (CFU) replating [68]. Interestingly, the same group recently reported comparing *DNMT3A* or *DNMT3B* single or double knockout (DKO) HSC in serial BM transplantations. They showed that DNMT3B plays a critical role in enabling HSC differentiation in the absence of *DNMT3A*. Although loss of *DNMT3A* alone has a more dramatic effect in HSC differentiation and overall the phenotypes of DNMT3A KO and DKO HSCs are predominantly similar, suggesting that DNMT3A has a more relevant function in regulating hematopoiesis [67].

Regulating DNA methylation is considered to be what shapes the topography of HSC differentiation [69]. Izzo and colleagues showed that DNMT3A KO HSCs present with transcriptional priming towards specific hematopoietic lineages in uncommitted HSCs [69]. DNMT3A null HSCs display reduced monocytic clusters (i.e., reduced Ly6c2, Prtn2 and Lyz2 expression) and skewing toward the erythroid lineage, as evidenced by increased Car1 and Car2 expression, although RBC levels remained unchanged. Furthermore, changes in DNA methylation also occurred in TF binding sites, evidenced by decreased activity in CpG-rich erythroid TF motifs in DNMT3A KO HSCs. Additionally, this study confirmed stem-cell priming from human DNMT3A-driven CHIP by isolating circulating CD34+ cells from patients. Sequencing data reconfirmed reduced monocytic priming and increased erythroid priming, as evidenced by increased GATA-1+ progenitors. The precise mechanism of HSC priming still remains largely unknown.

Interestingly, a paper in PNAS reported opposing results utilizing DNMT3A R878H mice instead of DNMT3A KO mice [70]. These mice mimic human DNMT3A-driven CHIP by modelling the most common R882 mutation in CHIP, MDS and AML. The DNMT3A R882 mutation has been proposed to act as a dominant negative by most [71,72,73,74], but not all groups [75]. The R882 mutation has also been proposed to alter the flanking sequence preference of DNMT3A [75,76], as well as sequester WT DNMT3a [77]. Using a preclinical model of DNMT3a-R882, it was shown that these mice present with a myelomonocytic type of AML, evidenced by increased circulating WBCs, particularly monocytes and platelets, as well as immature hematopoietic cells in the BM, blood and spleen (Figure 2A). Methylated DNA immunoprecipitation sequencing revealed both hypermethylated and hypomethylated DNA segments in mature myeloid leukocytes, which decreased Gata2, Gata3 and Pax5 and increased Rpl22, Eif4a1 and mTOR expression. LSKs also displayed increased mTOR, as well as CDK1, which is important in the cell cycle. Indeed, LSK hyperproliferation in DNMT3A R878H mice was attributed CDK1-mediated phosphorylation of EZH2 which inhibited the tri-methylation of histone H2K27 (H2K27me3).

Taken together, these contradictory studies highlight the divergent effects of genetic loss of DNMT3a compared with R882 and non-R882 mutations in the blood (Figure 2A). The location of the mutation appears to heavily influence HSC cellular functions, lineage skewing, WBC production and likelihood for leukemic transformations. Indeed, DNMT3A mutations at R882 show the most severe AML transformation potential, whereas other DNMT3A mutations are far less likely to progress [60].

### 4.2. TET2

TET2 de-methylates DNA and is one of the most commonly mutated genes in both MDS and CHIP, as well as CMML. It was the first gene reported to exhibit somatic mutations in blood cells in CHIP patients (i.e., without leukemia) and more than 130 different *TET2* mutations have been reported in cancer-free CHIP patients [13,15,16,17,19,62,65]. A meta-analysis of six major CHIP studies revealed that 9% of healthy individuals have CHIP and more importantly 11–15% of CHIP is due to *TET2* deficiency [34]. Notably, the presence of ancestral TET2 mutation (mostly biallelic) is a crucial factor of the MDS pathophysiology, and it is likely derived from TET2 CHIP [34].

TET2 dysfunction appears to skew hematopoiesis towards the myeloid lineage (Figure 2B) [69]. Indeed, elegant studies using single-cell sequencing by Izzo and colleagues showed that HSCs deficient in TET2 present with transcriptional priming even in uncommitted HSCs [69]. TET-deficient HSCs displayed an enrichment of monocytic clusters, defined by Ly6c2, Prtn2 and Lyz2, which was accompanied by a reduction toward erythroid priming (i.e., reduced Car1 and Car2 expression). Interestingly, the methylation changes observed in TET2 KO HSCs were commonly found in CpG-rich motifs within known transcription factor binding motifs. These data suggest that the HSC hyperproliferation and myeloid skewing in TET2 deficient cells are due to alterations in transcription factor regulation [69]. Furthermore, Moran-Crusio et al. showed that specific hematopoietic TET2 loss of function promotes HSC self-renewal, causing a dramatic competitive growth advantage over time [20]. In addition, TET2 loss-of-function mutations are mostly heterozygous, which result in DNA hypermethylation, HSCs gene dysregulation, and aberrant myeloid-specific proliferation [94,95].

However, it is important to note that most studies describing TET2 function in hematopoiesis reflect more on the role of TET2 but not what is actually occurring during CHIP. TET2-driven CHIP, as well as MDS, is actually characterized by TET2 loss-of-function rather than TET2 protein deletion, and in order to compare these differences, Ito’s group created mice carrying a mutation specifically in the catalytic domain (C terminal) of TET2 and compared these mice to the TET2 KO mice. They showed that TET2 catalytic mutant mice exhibited clonal outgrowth solely towards myeloid lineage, whereas TET2 KO mice presented an increase in either myeloid or lymphoid cells (Figure 2B) [79]. This demonstrates the difference between TET2 deletion and TET2 dysfunction, revealing a more translational model of TET2-CHIP. Mechanistically, the mutation in the catalytic domain interferes with Fe^2+^ and/or α-ketoglutarate (α-KG) binding, which is essential for TET2 function, resulting in impaired 5mC oxidation and DNA hypermethylation [96].

Interestingly, an elegant discovery by the Aifantis group showed that Vitamin C can restore TET2 activity acting as a nutraceutical mimic of TET2 activation. Specifically, vitamin C operates as a cofactor for Fe^2+^ and α-KG-dependent dioxygenases, activating the catalytic domain and enhancing 5 hmC formation in TET2-deficient HSCs [97]. Additionally, another important finding showed that NAD-dependent histone deacetylase-1 (SIRT1) transduces epigenetic changes in TET2, enhancing its functionality. Through RNAi screening and proteomics analysis, it was revealed that SIRT1 deacetylates TET2 at conserved lysine residues in the catalytic domain and enhances TET2 activity in cells that mimic TET2 mutant MDS cells [98].

#### *DNMT3A* and *TET2* Co-Deletion

Intriguingly, it may be reasonable to assume that the co-deletion of both *DNMT3A* and *TET2* may not provoke further hematopoietic consequences compared to *DNMT3A* KO alone, given the dependence of *TET2* de-methylation on the methyltransferase activity of *DNMT3A*. However, *DNMT3A*/*TET2* co-deletion in mice had an accumulative effect. The *DNMT3a*/*TET2* DKO led to an AML-like phenotype, evidenced by increased BM HSCs, myeloid skewing, elevated circulating WBCs, anemia and increased immune cell infiltrate into other organs such as the liver, spleen and lungs [68]. DKO mice all died by 1 year, whereas 70% of either *DNMT3A* KO or *TET2* KO animals were still alive at the 1-year time point. The authors noted increased *Klf1* and *Epor* expression in HSCs of DKO mice, which drove cellular self-renewal by activating the JAK2-STAT5 signaling pathway. They also observed increased *Ikzf1, Ebf1, Cebpa* and *Cebpe* expression, which was associated with changes in both 5 mc and 5 hmc, but stronger with the latter.

### 4.3. P53

The TP53 gene, which encodes the tumor suppressor protein p53, ranks in the top five genes mutated in CHIP. Importantly, p53 is a transcription factor that regulates a large number of genes in response to a variety of cellular changes such as oncogene activation, DNA damage and inflammation [99,100]. Over the past decade, somatic TP53 mutations were identified in CHIP, as well as in hematological-malignancies-related therapies such as prior exposure to radiotherapy and chemotherapy [15,17,19,101,102]. In the hematopoietic lineage, p53 plays an important role maintaining HSC quiescence, specifically targeting the melanoma antigen family member Necdin regulating DNA damage response in HSCs [80,81]. Mutant p53 enhances the repopulating potential of HSCs, suggesting that p53 is highly involved in the regulation of HSC self-renewal (Figure 2C) [82,83,84]. Additionally, Chen and colleagues discovered an epigenetic pathway by which mutant p53 drives CHIP. Mechanistically, they have shown the mutant p53 interacts with the histone-lysine N-methylatransferase EZH2. The interaction enhances EZH2 chromatin association, thereby increasing the levels of H3K27me3, leading to genetic regulation of HSPC self-renewal and differentiation related genes [85].

Interestingly, mutations of EZH2 and p53 are observed in 10% and 20% of patients with MDS, respectively; therefore, the levels of DNA tri-methylation may be compromised in MDS [26,29,38,39,103,104,105]. Additionally, 30% of patients with t-MDS previously exposed to cancer therapies develop mutations in P53 as mechanism to evade chemotherapy-induced cell death [106,107]. Thus, the literature suggests that P53 mutation has become a potential target in clonal abnormalities (especially CHIP) due to its role in HSC fitness and its frequency in t-MDS [82,108].

### 4.4. ASXL1

ASXL1 is a mammalian homolog of the Drosophila additional sex combs family (1–3) and has a crucial role activating and suppressing the Hox genes (a set of transcription factor genes) [109,110]. Importantly, ASXL1 is mutated in patients with entire spectrum of myeloid malignancies including 21% of MDS and 25% AML patients [87,88]. As mentioned before, DNA methylation plays a pivotal role in hematopoiesis through the regulation of gene expression. Consistent with mutant DNMT3a and TET2, ASXL1 mutations are also involved in disordered hematopoiesis leading to increased HSC self-renewal, impaired differentiation and aberrant proliferation of HSCs (Figure 2D) [89].

Mutant ASXL1 disrupts epigenetic modifications, such as histone methylation or ubiquitination (H2AK199Ub, H3K4me3 and H3K27me3), which could contribute in the long-term to the development of CHIP [89,90,111,112,113]. The deletion of ASXL1 in mice facilitates aberrant gene expression through histone modifications resulting in myeloid transformation. Mechanistically, ASXL1 mutations disrupt polycomb repressive complex 2 (PRC2) in hematopoietic cells, causing a reduction in genome wide H3K27me3 occupancy. This altered epigenetic landscape leads to dysregulation of specific oncogenic target loci contributing to myeloid transformation and MDS. In fact, EZH2 is a catalytic component of PRC2 which catalyzes the tri-methylation of H3K27me3 and 10% of MDS patients harbor loss-of-function mutations in EZH2 [90,91]. These findings highlight the importance of EZH2 in normal functioning and provide mechanistic insight into how disruptions in ASXL1 contribute to MDS.

### 4.5. JAK2

JAK2^V617F^ is also a common driver of CHIP; ultra-deep sequencing data detected the mutation in 1% of cancer-free adults over 60 years of age [15,114]. Additionally, the V617F variant has been associated with a number of myeloid proliferative diseases [115]. Recently, Steensma et al. demonstrated that 5% of MDS patients had the JAK2^V617F^ tyrosine kinase mutation and that, importantly, the presence of this mutation is associated with a higher rate of death [86,92]. Mice carrying heterozygous JAK2^V617F^ mutations showed significant increase of red blood cells and expansion of HSCs, as well as myeloid progenitors in BM, suggesting an important role for JAK2^V617F^ in hematopoietic cell regulation (Figure 2E) [116,117].

Interestingly, DNMT3A^R882^ and JAK2^V617F^ mutations drive slow but inexorable clonal expansion, being more common along the process of ageing, but they are the only two mutations also found in young adults (30–39 years of age) [114]. Importantly, in BM transplanted mice carrying JAK2^V617F^ mutations Sano et al. discovered that mutant mice displayed increased expansion of monocytes and neutrophils in the blood [93]. In order to address the effect of JAK2^V617F^ mutations in myeloid cells, they transduced THP-1 (human monocytic cell line) cells with GFP expressing JAK2^V617F^ or JAK2^WT^ and found that mutant cells exhibited activation of STAT1 signaling (phosphorylation at Y701 and S727 sides). Furthermore, JAK2^V617F^ cells upon stimulation with LPS showed significant up-regulation of inflammatory markers such as IL-6, IL-1β, TNF and CCL2, suggesting that JAK2^V617F^ cells have a heightened inflammatory response, which is linked to an increase in cardiovascular disease [93].

## 5. CHIP—A Novel Connection between CVD and MDS

Atherosclerotic CVD and leukemia are both responsible for vast global mortality, and while the etiology of these two diseases was historically thought to be mutually exclusive, the recent discovery of CHIP provides genetic evidence that an overlap between the two exists. While it is well-known that CVD is accelerated by metabolic disorders such as diabetes and obesity [118,119,120], as well as autoimmune diseases such as rheumatoid arthritis [121], there is emerging evidence that CHIP-related mutations found in MDS also contribute to heightened CVD in patients with myeloid neoplasms such as MDS [3,122,123]. Indeed, CVD was recently shown to be the most common non-disease related cause of death in MDS patients [3,122,124]. A matched cohort study of Surveillance, Epidemiology, and End Results (SEER) suggested that, in older adults, MDS is not only an independent risk factor for CVD but also highly associated with mortality in MDS patients [123]. Moreover, MDS diagnosis confers a significant risk especially for myocardial infarction (MI), even in relatively healthy older adults with minimal additional cardiovascular comorbidities. The underlying mechanism behind the increased prevalence of CVD in MDS is not clear; however, recurrent somatic mutations in the CHIP-related genes DNMT3A, TET2, JAK2 and AXSL1 may contribute to the cardiometabolic burden.

Both animal and clinical studies have revealed a causal relationship between CHIP and CVD, with a focus on DNMT3A- and TET-2-driven mutations (mainly due to the availability of animal models and these genes accounting for the majority of the annotated mutations) (Figure 2) [13,14,17,18,24,78,79]. One of the hallmarks of the atherosclerotic CVD is the macrophage infiltration in the intimal walls of the aorta, leading to macrophage lipid uptake and transformation into foam cells. Importantly, these cells are derived from circulating monocytes, which are well established to directly increase the risk of CVD [125]. Previous work investigating enhanced hematopoiesis in various inflammatory contexts has largely concluded that the increased number of circulating myeloid cells, namely monocytes, neutrophils and platelets, directly exacerbates atherosclerotic CVD. Conversely, although CHIP enhances CVD, circulating WBC numbers remain largely unchanged [15,17].

In order to understand how these somatic mutations drive CVD, Fuster and colleagues induced murine TET2-driven CHIP in an atherosclerotic mouse model (Figure 2B) [17]. They performed a competitive BM transplant (BMT), using 10% Tet2^−/−^ BM and 90% WT littermate control BM into the atherosclerotic mouse model: the low-density lipoprotein receptor KO mouse (Ldlr^−/−^ mice). In this study, mice with murine TET2-CHIP exhibited almost twice the atheroma size in the descending aorta, as well as increased lesion size and complexity (i.e., increased macrophage infiltration) in the aortic sinus. Notably, the authors attributed these atherogenic consequences in murine CHIP to expanded TET2 mutant clones and macrophage-driven IL-1β production in lesions [17]. While the importance of IL-1β in this context is still controversial, it has been suggested that TET2 may be a negative transcriptional regulator in response to inflammation. Macrophages deficient in TET2 upregulate a multitude of inflammatory chemokines, cytokines and their receptors, and mice with Tet2^−/−^ BM had elevated chemokines important in monocyte adhesion and recruitment such as CXCL1, CXCL2 and CXCL3 [15]. Similarly, an elegant study by Sano et al. used a CRISPR approach to generate HSC gene edited mice carrying TET2 and DNMT3A deficiency to investigate CVD in CHIP. They also used the BMT technique ratio 1:9 to generate murine CHIP and confirmed that Tet2 gene disruption confers a competitive advantage to HSCs leading to cardiac dysfunction via IL-1β and IL-6 (Figure 2A,B). In parallel, they showed for the first time that DNMT3A gene disruption likewise promotes cardiac dysfunction. However, the nature of the inflammatory response differed, with higher CXCL1 and CXCL2 but not IL-1β [78].

Interestingly, ~20% of MDS patients harbor TET2 mutations and among other myeloproliferative disorders this mutation can occasionally predict JAK2 deficiency [126]. In light of the gene overlap between MDS and CHIP, JAK2^V617F^ mutation was another CHIP-MDS driver gene also linked to CVD development, as it is well-known to cause essential thrombocythemia and promote atherothrombotic vascular disease (Figure 2E) [93,125]. Consistent with previous observations of greater cardiometabolic risk in mice carrying Tet2 and Dnmt3a mutations, a recent investigation evaluated the fitness of HSCs expressing the JAK2^V617F^ transgene. The authors observed that JAK2^V617F^ hematopoietic cells had clonal expansion with similar kinetics to Tet2 mutations but much more robust than what was observed in Dnmt3a mutants [13,78]. Additionally, they found that JAK2^V617F^ mutations lead to the expansion of mutant clones specifically towards the myeloid lineage. Furthermore, using a specific system to restrict JAK2^V617F^ expression in myeloid cells in a model of heart failure, mice carrying JAK2^V617F^ cells were more susceptible to cardiometabolic complications [93]. JAK2^V617F^ also activates an inflammatory cascade through STAT1 phosphorylation, which promotes MDS, CHIP and atherogenesis [14,93,127].

Taken together, these data suggest that the most common clonal mutations observed in MDS and CHIP are able to accelerate clonal outgrowth leading to CVD particularly atherosclerosis, MI and heart failure. This is significant as the individuals with CHIP or MDS who may not progress towards a leukemic transformation, should be screened for the risk of developing CV complications. This is particularly important in MDS patients with an existing history of CVD, such as a previous MI, where CHIP mutations are 5× more prevalent in these patients [14].

## 6. Conclusions

MDS is a complex disorder with a plethora of genetic mutations and abnormalities. Recent advances in next-generation sequencing have revealed specific gene mutations that have now been linked to different disease progression rates and diverse clinical outcomes in MDS/AML. Moreover, the recent discovery of the pre-MDS condition, CHIP, highlights that even before circulating leukocyte levels begin to change, these mutations in CHIP initiate HSC priming and alter their activity preceding malignant transformation of hematopoietic cells. While there are no current therapeutic avenues for patients that are diagnosed with CHIP, future work should focus on discovering what factors can drive or suppress clonal outgrowth. The current literature suggests that patients with low VAFs have a low-risk of developing CHIP complications, and thus future therapeutics that prevent mutated HSCs from outgrowing would limit the development of CHIP-related co-morbidities. Furthermore, the acquisition of CHIP mutations has not only been linked to malignant transformations, but also to exacerbated CVD, and therefore patients should also be evaluated for potential CV complications.

## Figures and Tables

**Table 1:** MDS, CCUS and CHIP diagnostic criteria.

Diagnostic Criteria for MDS and Pre-Cursor Conditions (Defined by the WHO) [1,7]	
MDS	Persistent cytopenia in one or more peripheral-blood cell lineages and morphologic dysplasia (>10% dysplastic cells) in one or more bone marrow lineages on the basis of morphologic and cytogenetic abnormalities
MDS categories	MDS with: ● Single-lineage dysplasia ● Multilineage dysplasia ● Ring sideroblasts and single-lineage dysplasia or multilineage dysplasia ● Isolated del (5q) ● Excess blasts type 1 or type 2 ● Unclassifiable
CCUS	Unexplained cytopenia in one or more peripheral blood cell lineages; a somatic mutation at a variant allele frequency of at least 20% in one or more genes that are recurrently mutated in myeloid neoplasms and insufficient dysplasia (<10%) for an MDS diagnosis
CHIP	Normal peripheral blood cell counts with a somatic mutation at a variant allele frequency of at least 2% in a gene that is recurrently mutated in myeloid neoplasms

**Table 2:** Summary of the generic causes of MDS discussed in this review.

Summary of the Genetic Causes of MDS	
Somatic Mutations	
Epigenetic Regulators	*TET2, DNMT3A, ASXL1, EZH2* genes
RNA Spliceosome	*SF3B1, SRSF2, U2AF1* genes
DNA Transcription	*RUNX1, TP53* genes
Signal Transduction Pathways	*KRAS, NRAS, JAK2* genes
Cohesion complex	*SMC3, SMC1A, RAD21, STAG2* genes
Germline Mutations	
*CEBPA, DDX41, ETV6, GATA2, RUNX1* genes	
Inherited Disorders	
Fanconi Anemia	*FANC* genes
Shwachman-Diamond Syndrome	*SBDS* genes
Li-Fraumeni Syndrome	*TP53* gene
Diamond-Blackfan Amemia	*GATA1/RPS19* genes
Dyskeratosis congenita	Telomerase complex disorder
Chromosomal Abnormalities	
Chromosomal Deletion	del(5q), del(7q), del(20q), del(17q)
Mosaicism syndromes	Trisomy 8

## Data Availability

No new data were created or analyzed in this study. Data sharing not applicable.
